# Penetrating Head Injury by a Hit of Rake in a Child: A Case Report and Literature Review

**DOI:** 10.1155/2023/9921985

**Published:** 2023-11-18

**Authors:** Issa Ibrahim Assoumane, Nicaise Kpègnon Agada, Rabiou Maman Sani, Aminath Kélani

**Affiliations:** ^1^Niamey National Hospital, Niamey, Niger; ^2^Faculty of Health Sciences of Abdou Moumouni University of Niamey, Niamey, Niger; ^3^Zinder National Hospital, Zinder, Niger; ^4^Faculty of Health Sciences of University of Zinder, Zinder, Niger

## Abstract

**Background:**

A penetrating head injury (PHI) refers to a situation where a projectile has breached the cranium but does not exit it. It constitutes about 0.4% of all head injuries. Several nonmissile materials inserting the skull have been reported. But to our knowledge, never before has any case of PHI caused by a hit of rake been reported. We report a first case of PHI caused by a rake in a child; then, we relate our experience with its management and discuss the relevant literature. *Cases Description*. A 5-year-old boy has been admitted with a rake embedded in his head. That occurred during a violent play with a neighbor. At presentation, the child was alert; there was no neurological deficit. The rake was embedded in the parietal regions on each side of the midline. The head Computed Tomography (CT) scan performed showed a biparietal hyperdensity from either side of the midline with a metal artifact. In the operating room, after a transversal incision joining the 2 tips of the object, we performed successively bone flaps; object extraction; debridement; duraplasty; and closing. The outcome was uneventful.

**Conclusion:**

This is the first case of PHI by a rake. The surgical management constitutes the main challenging point.

## 1. Introduction

Based on the mechanism, head injuries are classified into nonpenetrating or penetrating types [1]. The latter may be caused by a missile or nonmissile object [1–7]. In addition, nonmissile penetrating injuries are classified into 2 types: those entering through a natural orifice (orbit, nose, mouth, or ear) and those whose object really crosses the skull, causing a fracture and creating an artificial orifice [2]. A penetrating head injury (PHI) refers to a situation where a projectile has breached the cranium but does not exit it [1, 8]. PHI constitutes about 0.4% of all head injuries [1, 2, 4, 6, 9], where the foreign body produces injury to the brain in about 10% of cases [1, 10]. Through the literature, the reported nonmissile materials penetrating the skull include nails, metallic screws, stones, metallic rods, wooden sticks, chopsticks, pencils, knives, scissors, arrows, and other objects with a sharp edge [3, 6, 9, 11]. Nonmissile PHIs are characterized by a low-velocity impact of <100 m/s and cause damage by laceration and maceration, unlike missile injury that are damaged by kinetic and thermal energy [1, 2, 6]. In nonmissile-related injuries, brain tissue can be damaged and the degree of this damage is determined by a number of factors such as the properties of the penetrating object (type, velocity, size, etc.) [1, 6], the characteristics of the involved tissues (skull, muscle, mucosa, etc.), the angle of approach, the site and depth of the initial injury, anatomic, and neurovascular structure of the passage, and the presence of any secondary projectiles, such as bone or metallic fragments [6]. Some complications can occur after PHIs, such as meningitis, abscess, seizures, pneumocephalus, vascular laceration or occlusion with hematoma, aneurysm, pseudoaneurysm, and carotid-cavernous fistula [1, 11].

Head CT scan constitutes the imaging of choice for evaluating any penetrating cerebral trauma [1, 12]. It helps in the localization of the projectile, any fragments, bony destruction, in-driven debris, and identification of any secondary associated lesions [1]. In cases of doubt about vascular injury, cerebral angiography should be performed [1, 12].

So far, the initial management of patients with PHI remains so diverse [9], certainly because of the variety of implied objects and, in parallel, the variety of secondary lesions that can occur. Due to this, the preoperative neuroradiologic assessment is paramount for the correct neurosurgical approach [2].

In this article, we report a case of PHI caused by a hit of rake in a 5-year-old boy treated in the neurosurgical department of the National Hospital of Niamey. Through this report, we aimed to relate our experience with the management of this kind of PHI and discuss the relevant literature about it.

## 2. Case Presentation

**Figure 1:**
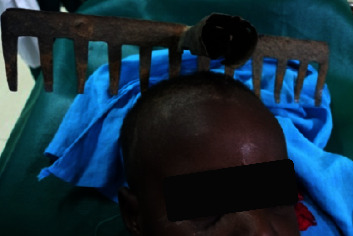
Preoperative image showing the rake embedded in the parietal regions.

**Figure 2:**
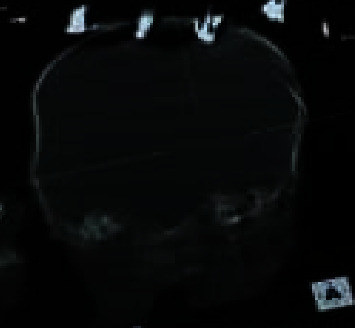
Bone window head CT scan in coronal section showing 2 hyperdensities from either side the midline with metal artifact.

**Figure 3:**
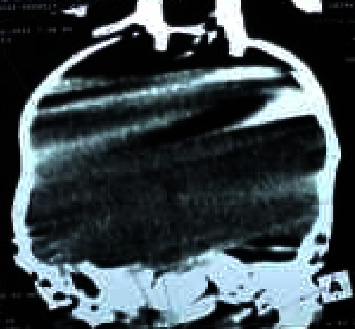
Parenchymal window head CT scan in coronal section showing 2 hyperdensities from either side the midline with metal artifact.

A 5-year-old boy was admitted with a rake embedded in the head (Figure 1). That occurred during a violent play with a neighbor; he was admitted less than 4 hours after the trauma. There was no notion of loss of consciousness, seizure, neurological deficit, or signs suggesting raised intracranial pressure. At presentation, he was found to have stable vital signs and was conscious and oriented in person, place, and time. He was not pale, febrile; he had no obvious distress signs. The rake was embedded in the parietal bones on each side of the midline with some bleeding from the entry points but no cerebrospinal fluid (CSF) leakage and no bleeding from craniofacial orifices. The head CT scan performed showed parietal hyperdensity from either side of the midline with metal artifact (Figures 2 and 3). So, it indicated foreign body extraction. The preoperative investigation performed was normal. Priory, antibiotic prophylaxis covering gram-positive, gram-negative, and anaerobic bacteria was commenced. He was given tetanus protection.

**Figure 4:**
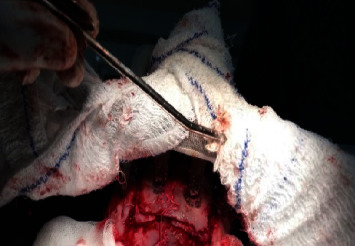
Intraoperative image showing the bone flap centered on the 2 penetrating points of the 2 tips of the rake; the bony filing at the base of the 2 penetrating tips is visible.

**Figure 5:**
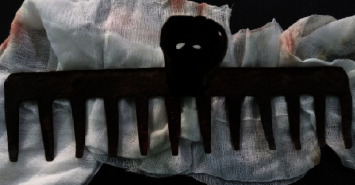
The extracted rake.

**Figure 6:**
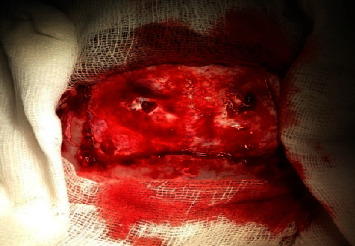
Intraoperative image showing the 2 dural breaches corresponding to the 2 entry points of the 2 tips.

**Figure 7:**
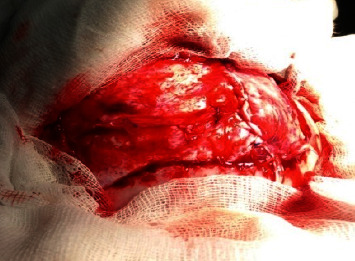
Intraoperative image taken after duraplasty.

In the operating room, under general anesthesia with a cuffed endotracheal tube in situ and a strict and sterile condition, we performed a transversal incision joining the 2 penetrating tips of the rake and extended it exteriorly. Then, we proceeded with subcutaneous dissection with parietal bones exposition. A bone flap was performed followed by a bony filing at the base of the 2 tips (Figure 4). And then, we proceeded with the bone flap extraction together with the two tips of the rake (Figure 5). That allowed us to discover 2 punctiform breaches on the dura mater at the penetrating points of the rake (Figure 6). Then, wound debridement was performed followed by duraplasty using two pieces of galea that we fixed over each dura breach with a 4-0 prolene (Figure 7); then we tented the dura, replaced, and fixed the bone flap; and finally we performed a layer and layer closing of the subcutaneous and cutaneous tissues followed by a bandage.

Postoperatively, antibiotics (intravenous ceftriaxone and metronidazole, then oral amoxicillin/clavulanic acid and metronidazole) were continued for 14 days. No postoperative complications such as seizure, neurological deficit, visual disturbance, and epistaxis leakage were noted. Wound stitches were removed on day 10, and he was discharged home. Seen successively 1 and 3 months later, he was still in good condition.

## 3. Discussion

### 3.1. Epidemiology

PHIs account for a rare condition with an incidence reported to be 0.4% of all brain injuries [4, 9]. Among this small percentage, children are more at risk of PHIs because of their softer skulls [9, 13] and also because of their disturbance and willpower to play with everything. Regarding the entry sites, the orbit and temporal region are the most commonly reported with orbitocranial penetrating injuries accounting for up to 45% of overall PHIs in children [9]. Some authors reported other uncommon sites such as the frontal region [14]. Considering the offending objects, many and various types have been reported: iron rod, wood, bamboo, stone, scissors, arrow, chopsticks, pen, nail, and harpoon [14]. In our case, the entrance site was through the parietal regions bilaterally, which is less common, and the material was a rake. This case of PHI caused by a hit of rake constitutes the first through the literature in our knowledge. Either in adults or in children, the male sex is reported to be predominant (>80%) [15], with a variable age [9]. Indeed, little boys seemed to be more involved in violent games than little girls. According to the literature, falls on sharp objects or accidents during play constitute the most common causes of PHIs [9, 11]. That was the case with our patient where the accident occurred during a violent play with a neighbor. However, other authors reported aggression [9, 15] or domestic violence and child abuse [9, 16] as occurring contexts as well. Therefore, nonaccidental injuries in children must be kept in mind [9, 13].

### 3.2. Clinic

In some reported case series, most patients with nonmissile PHIs have a good clinical condition with clear consciousness state at admission [2, 9]. But other authors reported the inverse results [4]. Anyway, we can say that the initial clinical condition is very variable and strongly depends on the severity of the injury, the features of the offending material, and the extent of the damage caused by the material.

### 3.3. Neuroimaging

Initial examination includes a plain skull X-ray which can show the penetrating object, the existence of skull fractures, and the advantage of being free of metallic artifacts [2, 14]. Then, a brain CT scan and a brain magnetic resonance imaging(MRI) can be performed to analyze the brain parenchyma. Through the bone window on a CT scan, bone injuries will be more assessed, and with the soft window, the relation of the object to surrounding anatomical structures will be analyzed and secondary associated brain injuries will be ruled out [4, 14]. In addition, a 3D-constructed CT scan can provide further valuable information about the object's size, length, direction, and position at various angles [14]. If vascular injury is suspected, noninvasive investigation with CT angiography (CTA) or conventional angiography in stable patients can be a good option to avoid unnecessary exploratory surgery [4]. Although MRI constitutes the better tool for brain parenchymal assessment, its use is limited because most of the objects found in patients with nonmissile penetrating cranial injuries are metallic, and MRI can cause some secondary lesions relative to the migration caused by the MRI magnetic field. MRI is indicated when the foreign body is a fragment of wood, with T1 being more sensitive than the T2 sequence [2]. Our patient had a metallic object in his skull and therefore was not referred for an MRI evaluation.

### 3.4. Management and Outcome

Because of a number of factors such as the object type and its entry site and trajectory through the skull, patient characteristics, and brain injury mechanism [4, 14], a standardized approach for object removal in nonmissile PHIs is difficult to establish. However, objects are commonly removed through a craniotomy which has the advantages of early visualization and protection of neurovascular structures, controlled object removal, accessible debridement of the devitalized brain tissue, associated lesions management and adequate dural repair if needed [2, 14]. In our case, we performed craniotomy before the removal of the penetrating tips of the rake; with the craniotomy, any bleeding source could be easily mastered. There are known early and late postoperative complications associated with nonmissile PHIs [9, 14]. Early complications include parenchymal contusions, tract hematoma, dural tears associated with cerebrospinal fluid leak, infection, direct blood vessels injury [11, 14, 17], and seizures [18]. Late complications include the development of pseudo-aneurysms, foreign body migration, arteriovenous fistula, and posttraumatic epilepsy [14, 19]. In our case, the patient did not develop any early or late postoperative complications.

The initial admission Glasgow Coma Scale(GCS) score, pupil size, and initial CT scan findings [14, 20] condition the outcome of the patients after nonmissile PHIs. Initial GCS <5 is usually associated with marked neurological function damage and poor prognosis. Brain stem involvement on the initial CT scan also has poor prognosis and is mostly fatal [9, 14]. In the present reported case, the initial GCS was 15, which is above the severity's cutoff point, and the initial CT scan had not shown any brainstem involvement, which could explain the uneventful outcome in our patient.

## 4. Conclusion

Pediatric nonmissile PHIs are rarer compared to adults and furthermore, this case due to a rake is very unusual and constitutes the first reported in the literature to our knowledge. Regarding the surgical technique, by filing the bone at the base of the 2 penetrating tips of the rake before removing these, we ensure ourselves to avoid any undesirable movement which could trigger more secondary intracranial damages. We think that surgical technique could help in the future if a similar case occurs.

## Data Availability

Data used in this study are available upon reasonable request from the corresponding author.
